# The perceptual learning of time-compressed speech: A comparison of training protocols with different levels of difficulty

**DOI:** 10.1371/journal.pone.0176488

**Published:** 2017-05-18

**Authors:** Yafit Gabay, Avi Karni, Karen Banai

**Affiliations:** 1 Department of Communications Sciences and Disorders, University of Haifa, Haifa, Israel; 2 Edmond J. Safra Brain Research Center for the Study of Learning Disabilities, Department of Learning Disabilities, University of Haifa, Haifa, Israel; 3 Department of Special Education, University of Haifa, Haifa, Israel; 4 Sagol Department of Neurobiology, University of Haifa, Haifa, Israel; Universitat Zurich, SWITZERLAND

## Abstract

Speech perception can improve substantially with practice (perceptual learning) even in adults. Here we compared the effects of four training protocols that differed in whether and how task difficulty was changed during a training session, in terms of the gains attained and the ability to apply (transfer) these gains to previously un-encountered items (tokens) and to different talkers. Participants trained in judging the semantic plausibility of sentences presented as time-compressed speech and were tested on their ability to reproduce, in writing, the target sentences; trail-by-trial feedback was afforded in all training conditions. In two conditions task difficulty (low or high compression) was kept constant throughout the training session, whereas in the other two conditions task difficulty was changed in an adaptive manner (incrementally from easy to difficult, or using a staircase procedure). Compared to a control group (no training), all four protocols resulted in significant post-training improvement in the ability to reproduce the trained sentences accurately. However, training in the constant-high-compression protocol elicited the smallest gains in deciphering and reproducing trained items and in reproducing novel, untrained, items after training. Overall, these results suggest that training procedures that start off with relatively little signal distortion (“easy” items, not far removed from standard speech) may be advantageous compared to conditions wherein severe distortions are presented to participants from the very beginning of the training session.

## Introduction

Our cognitive system is remarkably adaptive to changes in the environment. Speech highlights this issue because despite the traditional view that speech perception is a static process (e.g., [1]), many studies suggest that dynamic processes such as perceptual learning are inherently involved in various aspects of speech perception [2–5]. Specifically, the intelligibility of speech that is initially incomprehensible due to acoustic distortions or background noise improves substantially with different experiences (prior exposure, short-term practice, long-term use) with such speech. Many studies focused on the linguistic or the acoustic-phonetic aspects of learning from speech experiences [6–10]. Other studies focused on factors that are associated with aspects such as feedback, training length or the nature of the experience required to yield learning [11,12]. Nevertheless, the widely held assumption inherent to many training protocols that gradual changes in the level of difficulty during training promote learning, has not been systematically tested in the past. The major goal of the current study was therefore to compare the effects of four training protocols that differed in how (or whether) difficulty changed over the course of training on the perceptual learning of time-compressed speech and its generalization across tokens and talkers.

The prevailing approach to the study of various forms of speech learning is to provide relatively little training with little stimulus repetition [13–17]. For example, in studies on the perceptual learning of time-compressed speech, brief exposure to a set of time-compressed sentences yielded significant improvements in the perception of a new set of sentences [13,16,18]. In those studies, there was no stimulus repetition between training and testing or within the training materials. Therefore, the reported improvements reflect substantial generalization of learning. Nevertheless, there is no data to suggest that this brief exposure with no stimulus repetition yields optimal learning or generalization. For example, in the case of time-compressed speech, multi-session training yielded more learning and generalization than either brief exposure or a single training session [19]. However, even within a single session, training comprised 300 trials during which the rate of compression was adapted using a staircase procedure. After training, recognition of the trained materials was significantly more accurate in trained listeners than in listeners who received only brief exposure to time-compressed speech. However, only multi-session training yielded generalization to novel speech materials. These findings imply that longer training might broaden the scope of generalization, although the contribution of additional training trials might be smaller than that of initial exposure trials.

Another potential account of the differences in the scope of generalization across studies is that generalization is affected by changes in the level of acoustic distortion over training rather than by the absolute amount of training. Specifically, in brief exposure studies exposure was provided at fixed amount of compression (usually, but not always, the same as in the test). On the other hand, protocols in which the amount of distortion changes over time were used in multi-session training studies with time-compressed speech [3,19] as well as with other forms of distorted speech [10,20–22]. Therefore one of the goals of the present study was to compare adaptive and non-adaptive training protocols using otherwise identical procedures (e.g., in terms of training length, trained materials and the degree of stimulus repetition during training).

### Adaptive approaches

Adaptive procedures were found effective in improving the discrimination of many acoustic features (for reviews see, [23,24]), but the direct comparison of different procedures has been rare [25]. Adaptive procedures also induce significant learning distorted speech in the general population [10,22] as well as in different clinical populations [26–28].

There are several adaptive training protocols for skill learning. One method is to gradually increase the level of difficulty during training with a staircase procedure. The hallmark of such procedures is that the level of difficulty is adapted based on the trial-by-trial performance of individual participants, but subjects experience many moderate to high difficulty items [29–32]. Another training approach is weighted towards providing experience with items that are deemed to constitute relatively small deviation from familiar items (i.e., with a relatively easy task conditions). One such approach called “Errorless Learning”, is based on the assumption that errors that occur during training strengthen incorrect associations and are therefore detrimental to the learning process [33,34]. In these training protocols, the participant progresses from easy to difficult items and effort is being made to minimize [35] or avoid task conditions that may lead to errors [33]. Errorless training has been found to be an advantageous training method for memorizing words compared with training methods in which errors were encouraged by asking subjects to guess at the correct answer [36,37]. The main differences between staircase and "easy to difficult" training methods are in how fast participants get to difficult items and in how much time is spent on "easy items". In "easy to difficult" training protocols, the level of difficulty is changed at a pre-determined incremental pace independently of the participants’ level of performance and most (or all) of the training is on relatively high performance levels. In adaptive-staircase training, the level of difficulty is determined based on the participants’ performance on a few previous trials and is rapidly increased as long as no errors are made. Consequently, errors occur multiple times throughout training.

Some training approaches emphasize that for robust learning to occur, a desired level of difficulty (in terms of effort rather than performance level) should be experienced during training. In particular, it is suggested that more effortful retrieval evoked by more difficult conditions may lead to greater strengthening in the underlying memory trace than less effortful retrieval assuming that successful retrieval occurs in both situations [38]. Such “desirable difficulties” have been suggested to be triggered by, for example, the varying of the conditions of learning rather than keeping conditions constant and predictable, or by providing “contextual interference” during learning (e.g., *interleaving* rather than *blocking* practice). This approach was found beneficial in vocabulary learning [39], implicit sequential learning [40] and long-term memory [41].

Only few attempts were made to examine the influence of the training protocol on the perceptual learning of speech. Specifically Svirsky et al. [22] showed that gradual training can accelerate the adaptation to spectral alternations that typically render speech unintelligible. In addition Li et al. [42] showed that alternately exposing listeners to moderately-distorted and severely-distorted speech yielded greater learning of noise vocoded speech than exposing listeners to either of the distortions alone. However results are not always consistent and other studies showed that training with items with different levels of distortion was either detrimental [43] or had no influence on learning [21].

In the current study protocols in which the level of distortion of speech items changed gradually in an easy to difficult progression or in an adaptive-staircase procedure were compared to protocols in which the level of distortion was predetermined and fixed throughout training. We examined time-compressed speech learning following training on four protocols (1) constant low-compression: stimuli compressed to a single, pre-selected level that does not impede speech recognition will be presented throughout training; (2) constant-high compression: stimuli compressed to a single, pre-selected level known to severely impede speech intelligibility in naïve listeners will be presented throughout training; (3) adaptive-staircase: the level of compression will increase or decrease based on listeners' performance on previous trials; (4) adaptive-incremental: the level of compression will gradually increase across blocks of trails from easy to more difficult items in a pre-selected sequence. Based on the literature discussed above we hypothesize that training (on all protocols) should elicit more learning than participating in testing sessions alone. We also hypothesized that gradual exposure to increasing levels of distortion would benefit learning and thus that the adaptive protocols would elicit more learning (but perheps less generalization, e.g., [44,45]) compared with the constant protocols [38]. However, given the notion of errorless training one may expect that training in constant-easy conditions might be the more effective approach.

## Methods

### Participants

Sixty five native Hebrew speakers (ages 18–35, all undergraduate university students, 45 female, 20 male) without prior experience with psychoacoustic testing participated in the experiment. The male/female distribution was similar across groups with 3–5 males in each group (adaptive staircase; 5 males; constant-low compression, 4 males; control group 4 males; adaptive incremental 2 males; constant-high compression 3 males). By self-report, all participants had normal hearing and no history of language, learning or attention problems. In addition, in the recruitment process potential participants were explicitly asked if they were diagnosed with a learning disability (dyslexia or dysgraphia). Only participants who gave a negative response were included. Participants were compensated for their time. All aspects of the study were approved by the ethics committee of the Faculty of Social Welfare and Health Sciences at the University of Haifa (protocol 199/12) and written informed consent was obtained from all participants. Listeners were randomly divided into five groups who participated in no training (untrained controls), or were assigned to complete one of four training protocols: (1) Constant-low compression training (2) Constant-high compression training (3) Adaptive-staircase training (4) Adaptive-incremental training. All groups received feedback during training.

### Stimuli

All stimuli were recorded and sampled at 44 kHz using a standard microphone and PC soundcard by a young male native speaker of Hebrew (the trained speaker) using Audacity. In addition, a subset of the sentences was recorded by another native Hebrew speaker (also a young male) and used for tests of cross-talker generalization during the test phase. The naturally-spoken rate of the stimuli was relatively slow and resembled that of Israeli newscasters (and not daily conversational rate) [46]. The RMS levels of all sentences were normalized after recording and before compression. Stimuli were time-compressed using a WSOLA algorithm [47], which has been shown to modify the rate while preserving other qualities such as pitch and timbre. A total of 120 simple active subject-verb-object (SVO) sentences in Hebrew [48] (were used in this study. Each sentence was 5–6 words long and had adjectives modifying both the subject and the object. The naturally spoken sentences had an average duration of 3 seconds (range: 2.3–4.2 s) and an average rate of 109 words/minute (range 72–144). Sixty sentences were semantically plausible (true, e.g., “The municipal museum purchased the impressionistic painting”) whereas the remaining sentences (false) had a semantic violation which renders them implausible (e.g., “The municipal museum ate the impressionistic painting”). 100 sentences (50 true) were used for training. Twenty of those sentences were used in the pre-test and test phases to assess learning of the trained tokens. Likewise 20 of the trained sentences presented by a different talker were used to assess cross-talker generalization. The remaining 20 sentences were used to assess generalization to untrained tokens.

### Tasks and training protocols

**Fig 1:**
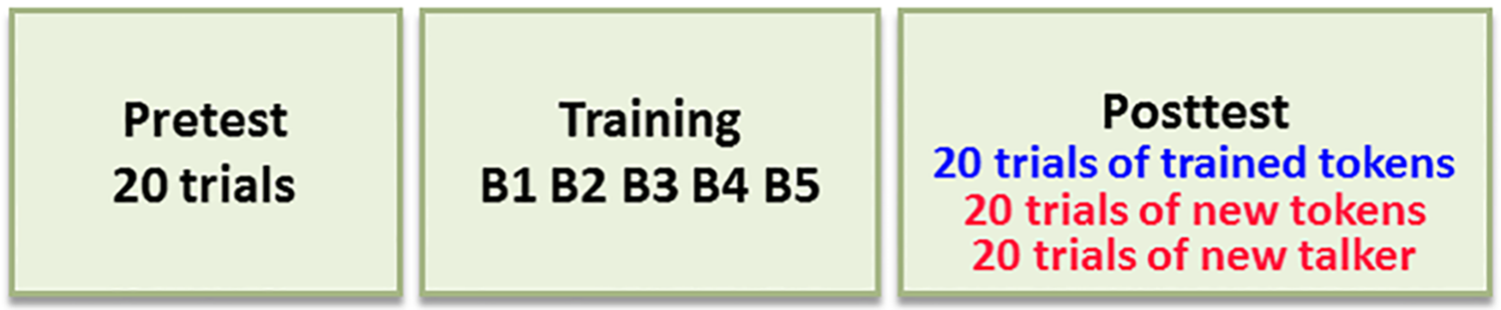
Procedure. Participants performed pre-test and five blocks of training on one of the protocols (each contained sixty trials) or no training. After that, participants performed a post-test with three conditions. Post-test performance on trained items is indicative of learning (marked in blue). Post-test performances with the new talker and with new tokens are indicative of generalization (marked in red).

During the pre-test phase, 20 sentences compressed to 30% of their naturally spoken duration were presented. During the post-test phase blocks of 20 sentences, compressed to 30% of their naturally spoken duration were presented. Participants were required to write them up as accurately as they could. The post-test phase included 3 different conditions of 20 trials each: (1) the repeated-tokens condition (20 sentences selected at random from the training set that were presented by the same male speaker of the training phase), (2) the new-tokens condition (20 new sentences with similar semantic structure that were presented by the same speaker of the training phase) and (3) the new-talker condition (20 sentences selected at random from the training set that were presented by a different male talker). The order of the three conditions was counterbalanced across participants. No feedback was provided during either the pre- or the post-test. The pre-and post-test phases were identical for all listeners. See Fig 1 for an illustration of the design.

The training set included 100 different time-compressed sentences. During training, listeners performed a semantic verification task on these sentences for 5 blocks of 60 trials. After hearing each sentence, listeners were required to determine whether it was semantically plausible (true) or not (false). Sentences were selected at random (without replacement) until all 100 sentences were presented, after which random selection started again. Visual feedback (smiling/sad face) was provided after each response. Each listener practiced with one of the following protocols:

1. constant-low compression protocol: stimuli were compressed to 60% of their naturally spoken duration, a level of compression that makes speech sound faster than normal but does not normally impede intelligibility in normal-hearing young adults.
2. constant-high compression protocol: speech was compressed to 30% of its naturally spoken duration, a level known to result in markedly reduced intelligibility.
3. adaptive-staircase protocol: training started with a compression level of 65% of the naturally-spoken duration. Subsequently, compression was adapted using a 2-down/1-up staircase procedure in 25 logarithmically equal steps to a maximal compression of 20% [49].
4. adaptive-incremental training protocol: Training started with a compression level of 60% in the first block, 50% in the second block, 40% in the third block, 30% in the second block and 20% in the last block.

The considerations that led us to select the stimuli (compression rates) were very similar to considerations behind many perceptual learning studies (e.g., [35]). The idea was to start from typical levels of performance and try to push the participants' performance as much as possible into conditions wherein untrained individuals would fail to discriminate or perceive the stimuli. Thus, the compression rates were chosen so as to provide experience with speech rates that are in the range of easily recognizable [46] up to highly speeded speech stimuli that could not be deciphered by native listeners without specific training [19,50]. The level of 20% compression was introduced because we expected that some individuals would be able to decipher compressed speech better than others and we wanted to challenge all participants to their limits in the adaptive conditions.

### Procedure

Testing on time-compressed speech tasks took place in a quiet room with participants seated directly in front of a computer monitor during the entire experiment. Stimulus presentation and manipulation were controlled by Matlab. Stimuli were presented binaurally using headphones (Sennheiser HD-215). The experiment had 3 phases: a pre-test phase in which baseline performance was assessed, a training phase (for the trained groups) and a post-test phase. During the pre-test and the post-test phases participants performed a self-paced sentence transcription task in which they had to write down each sentence they heard as accurately as they could. During the training phase, trained participant performed the semantic verification task described above.

The experiment was administered in one session of approximately one hour. Trained participants completed the pre-test, the training and the post-test phases whereas untrained participants completed the pre-test and the post-test phases only with 30 minutes break in between. Comparisons of trained and untrained listeners served to ascertain that post-test performance differences across groups are attributable to training rather than to exposure-induced learning associated with participating in the test phases.

### Data analysis

Performance during the pre-test and post-test phases was quantified as mean proportion of words correctly identified across all sentences of a given condition. In the analysis of data from these phases, orthographic errors (e.g., homophones) were not considered as errors because we were interested in how listeners heard the sentences and not in their writing skills. Incomplete/incorrect suffixes were considered as errors because in Semitic languages such as Hebrew they can have a big influence on the semantics of the sentence (e.g., changing the timing of an event from past to future).

Performance during the training phase was quantified using different indices of performance, depending on the protocol. For the constant (low and high compression) and adaptive-incremental training conditions, the mean proportion of sentences correctly verified on each block was used. Data from the training phase with the adaptive-staircase protocol is not reported here and interested readers are referred to published studies with the same stimuli [19,50]

To determine whether learning and generalization occurred data was analyzed as follows: First, baseline (pre-test) performance was compared across all groups to determine that naïve performance with time-compressed speech was equivalent groups. Subsequently, test-phase performance was compared across groups. Differences between trained and untrained listeners on the repeated-tokens condition were taken as evidence for learning. Differences on the new-tokens and new-talker conditions were taken as evidence of generalization. For each condition, a one-way ANOVA was conducted to determine whether significant group differences were observed. In addition additional post hoc analyses were conducted in order to determine whether (1) the trained groups differed from the control group, (2) the constant and the adaptive training protocols yielded different outcomes, and (3) whether within each protocol differences existed between the two groups that practiced with that protocol.

## Results

### Baseline performance

**Fig 2:**
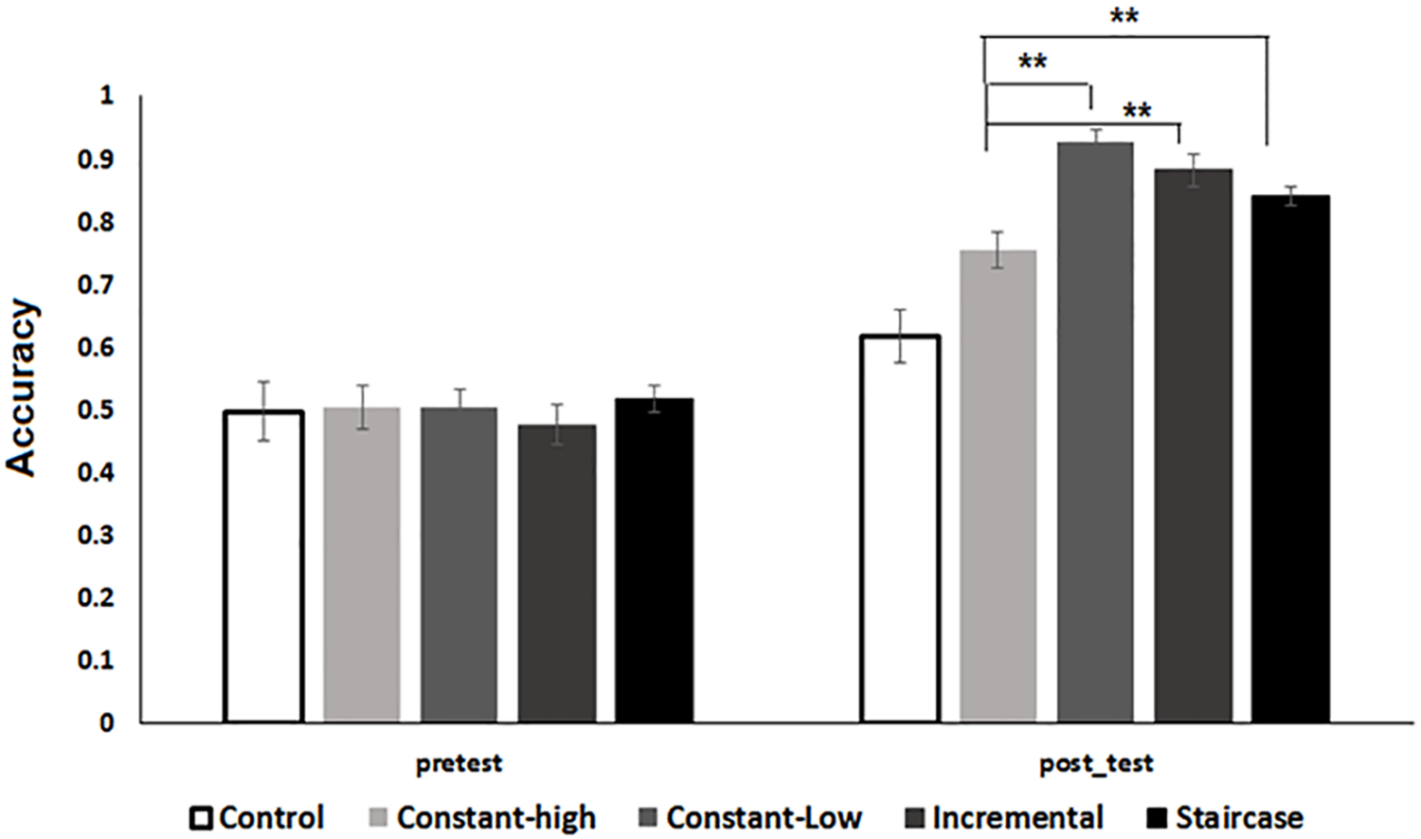
Pre-test and post-test performance on the trained condition as a function of group. Error bars show the 95% confidence interval of the mean. ** *p* < .01 in between group t-tests on post-test data.

The baseline (pre-test) performance of all groups is shown in Fig 2. An analysis of variance (ANOVA) with group (control, constant-low compression, constant- high compression, adaptive-staircase and adaptive-incremental) as a between subject factor and mean proportion of words correctly identified during the pre-test as the dependent variable showed no significant main effect of group (*F* (4, 60) = .21, *p* = .92, *η*_*p*_^*2*^ = .01), indicating that there were comparable levels of performance at baseline.

### Post-test performance

#### Performance on the repeated-tokens (trained) condition as a function of training and training protocol

As shown in Fig 2 and supported by a one way ANOVA, not all groups performed equivalently during the post-test phase (*F* (4, 60) = 21.9, *p* < 0.0001 .00, *η*_*p*_^*2*^ = .59). Moreover, planned comparisons revealed that all modes of training resulted in significant gains in performance compared to the no-training control condition (Adaptive staircase vs. control, *t* (24) = 5.23, *p* < .01); Constant-high compression vs. control, *t* (24) = 2.86, *p* < .01; adaptive incremental vs. control, *t* (24) = -5.66, *p* < .01; Constant-low compression vs. control, *t* (24) = -7.05, *p* < .01; significance after Bonferroni correction). A comparison between the two training protocol types, i.e., between the two constant and the two adaptive training protocols showed no significant differences (*t* (24) = 1.12, *p* = .27). There was no significant difference in the post-training performance of the two groups training in the adaptive conditions (*t* (24) = -1.49, p = .14). However, (as suggested by Fig 2) training in the constant-low compression protocol resulted in significantly better post-training performance compared to training in the constant-high compression protocol (*t* (24) = -5.38, *p* < .01). Indeed, training in the constant-high compression condition resulted in significantly smaller gains also compared to the two adaptive training conditions (Adaptive staircase vs. Constant-high compression, *t* (24) = 2.83, *p* < .01; Adaptive incremental vs. Constant-high compression, *t* (24) = -3.56, *p* < .01; Bonferroni correction). These results suggest that all four training protocols resulted in significant learning, i.e., improvement of the ability to decipher items that were previously encountered during practice. However, there was a significant advantage in training in either of the two adaptive protocols or in the constant-low compression training protocol compared to the training in the constant-high compression protocol, suggesting that the training procedures that started off with relatively little signal distortion were advantageous compared to a condition wherein severe distortions were presented to participants from the very beginning of the training session.

#### Generalization to new tokens

**Fig 3:**
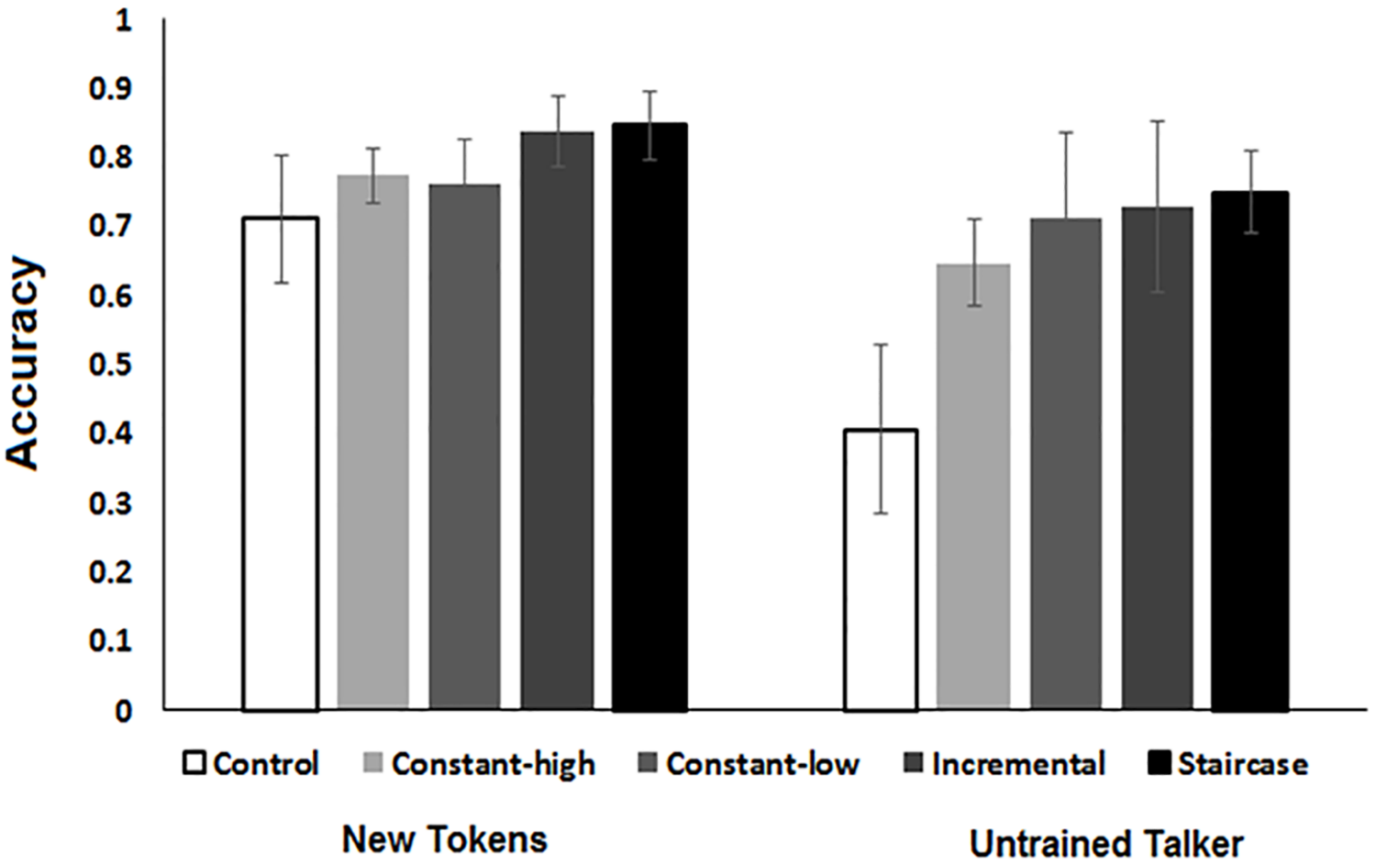
Posttests performance on the new tokens and new talker conditions as a function of training protocol. Error bars show the 95% confidence interval of the mean.

Post-training, compared to the no training control group’s performance in the new-tokens condition is shown in Fig 3. A one way ANOVA was conducted with group (control, constant-low compression, constant-high compression, adaptive-staircase and adaptive-incremental) as a between subject factor and mean proportion of word correctly reproduced in the new tokens condition as the dependent variable. The main effect of protocol was significant (*F*(4, 60) = 2.89, *p* = .02, *η*_*p*_^*2*^ = .16). Planned comparisons revealed that on average the trained groups scored higher than the control group (*F*(1, 60) = 5.2, *p* = .02, *η*_*p*_^*2*^ = .07). No significant differences were found between the two adaptive (*F*(1, 60) = .43, *p* = .51, *η*_*p*_^*2*^ = .006) or between the two constant training groups (*F*(1, 60) = .05, *p* = .81, *η*_*p*_^*2*^ = .009). Together, the two adaptive protocols elicited more generalization than the two constant training protocols (*F* (1, 60) = 5.83, p = .01, *η*_*p*_^*2*^ = .08).

#### Generalization to a new talker

Performance accuracy scores in the new-talker condition is shown in Fig 3. A one way ANOVA was conducted with group (control, constant-low compression, constant-high compression, adaptive-staircase and adaptive-incremental) as a between subject factor and mean proportion of word correctly identified on the new talker condition as the dependent variable. The main effect of group was significant, *F*(4, 60) = 7.57, *p <* .*001*, *η*_*p*_^*2*^ = .33). The planned comparisons revealed that on average trained groups differed significantly from the control group (*F*(1, 60) = 26.89, *p* = .00, *η*_*p*_^*2*^ = .308). Combined, the adaptive procedures did not elicit better performance than the constant procedures (*F*(1, 60) = 1.33, *p* = .24, *η*_*p*_^*2*^ = .02) and no significant differences were observed between the two adaptive (*F*(1, 60) = .11, *p* = .73, *η*_*p*_^*2*^ = .001) or the two constant training procedures, (*F*(1, 60) = .1.9, *p* = .16, *η*_*p*_^*2*^ = .03). To test the possibility that the constant-high compression condition differed from the three other training conditions (as suggested by Fig 3) an additional comparison (between the constant-high and the other 3 protocols) was run and showed a trend towards a significant difference (*F*(1, 48) = 3.57, *p* = .06), *η*_*p*_^*2*^ = .07), although the size of the effect was smaller than that of the other generalization effects that were presented so far.

Together, the results of the generalization tests show that relative to untrained controls training resulted in cross-token as well as cross-talker generalization. Whereas cross-talker generalization likely stemmed from the differences between the control group and all training protocols, cross-token generalization differences are partly attributable to a small advantage of the adaptive training groups.

### Training-phase learning in the constant and adaptive-incremental protocols

**Fig 4:**
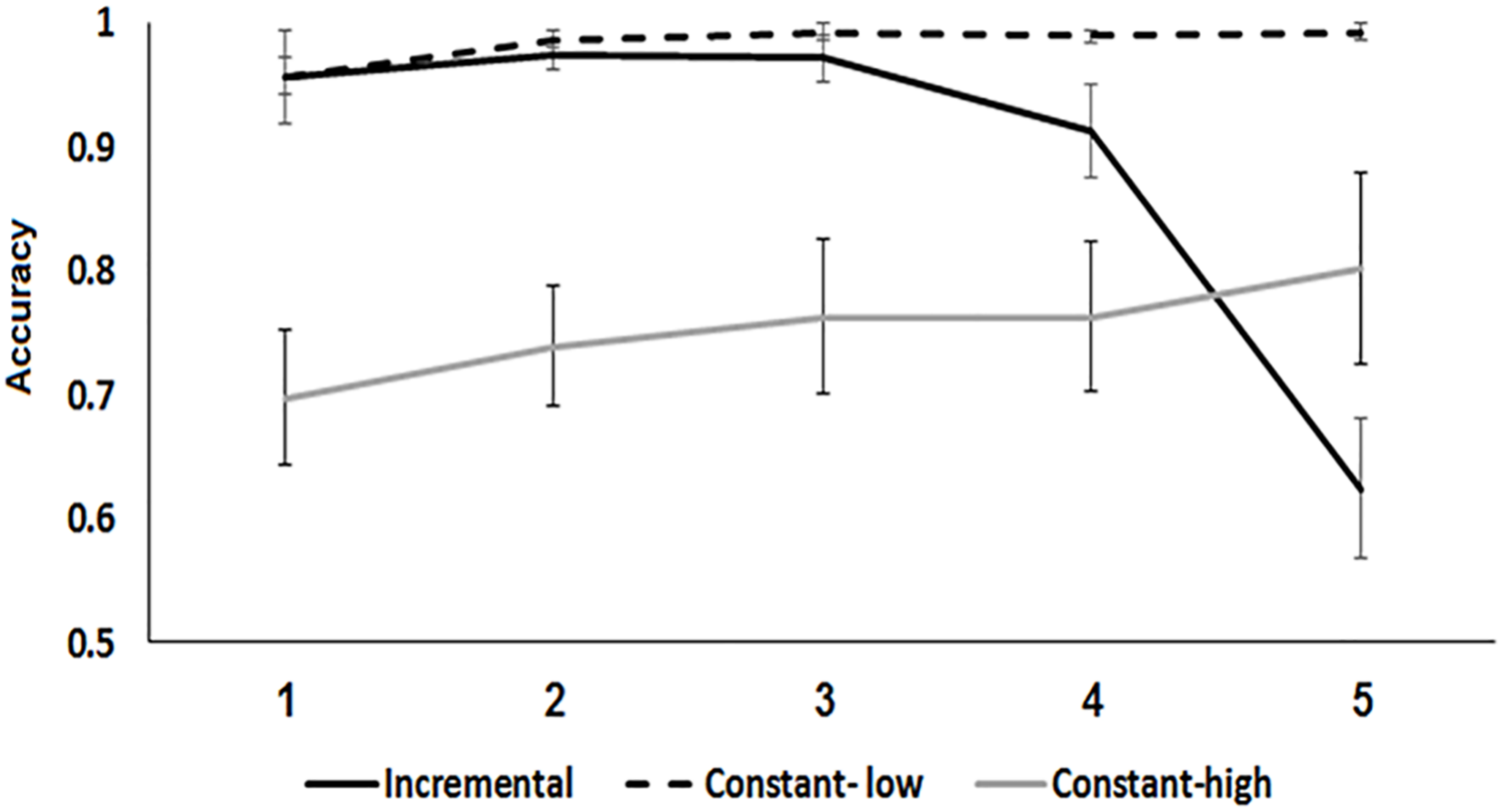
Training-phase performance of the constant-low compression, constant-high compression and adaptive-incremental groups as a function of training block. Note that while in the two constant conditions all 5 blocks were maintained at a single set level of task difficulty (level of compression 60% and 30%, in the constant-low compression and constant-high compression, respectively) in the adaptive-incremental condition task difficulty was increased across blocks (60%, 50%, 40%, 30%, 20%, blocks 1–5 respectively). Error bars show the 95% confidence interval of the mean.

A direct analysis of the learning curves of the adaptive-incremental group is not feasible; by definition, participants are expected to make very few errors during the initial training period, making it hard to document changes in performance accuracy. Furthermore, the training effect might be obscured by the decrease in performance accuracy that results from the greater perceptual challenge (increased level of compression) introduced in later blocks. Thus, to assess within-session learning differences, we compared performance accuracy at the 4^th^ training block of the session in which the level of compression was equal in the constant-high compression and the adaptive-incremental protocols. A one-way ANOVA was conducted with group (constant-high compression vs. adaptive-incremental) as a between subject factor and mean accuracy during the 4^th^ block (in which the level of compression was 30% in both groups) as the dependent variable. The results are presented in Fig 4. The main effect of group was significant (*F*(1, 24) = 17.32, *p* = .00, *η*_*p*_^*2*^ = .42), the adaptive-incremental training procedure resulting in greater accuracy (*M* = .91, S.E. = .02) than the constant high-compression (difficult) training procedure (*M* = .76, S.E. = .02). We also compared performance in the initial block of training (level of compression– 60%) in the constant-low compression and the adaptive-incremental training protocols. No significant differences were observed (F (1, 24) = .00, p = 1, *η*_*p*_^*2*^ = .0).

## Discussion

Speech encountered in many listening situations is hard to recognize due to distortions introduced by the talker (e.g., fast speech rates, foreign accents) or the environment (e.g., noise). Adapting to these perturbations is important and studies suggest that such adaptations involve perceptual learning if the distortion is reencountered a sufficient number of times [2–5]. In the present study we compared the effects of training regimens, that differed in how speech rate (time compressed) was changed across the training session, on the ability to perceptually disambiguate distorted speech. Five different groups were included in the study, an untrained control group and four trained groups: constant-low compression (single pre-selected easy level of distortion), constant-high compression (single pre-selected difficult level of distortion), adaptive-staircase (level of distortion changed based on participants’ performance on previous trials), and adaptive-incremental (level of distortion gradually increased from easy to difficult items in a pre-determined protocol). The results showed robust learning in all of the trained groups with all trained groups outperforming the un-trained control group on the repeated-tokens (trained) condition as well as on the new-talker condition.

Nevertheless, learning was least effective when training started and continued with highly compressed speech items (the constant-high compression protocol). Although listeners trained on this protocol performed better than untrained controls at the re-test, they also performed more poorly than listeners trained with the other protocols (Fig 2). This disadvantage was evident not only during the test phase, but also during training (Fig 4). Thus, after 3 blocks in which compression level increased gradually from a relatively non-challenging compression of 0.6 of the natural rate performance improved to a greater extent than after 3 blocks of highly compressed stimuli. Together, these findings suggest that initial training on "easier" items can facilitate the perceptual learning of time-compressed speech. An alternative possibility, that exposure to a large range of gradually increasing compression rates is the critical factor in facilitating learning seems less viable because the constant-low compression protocol was as effective as the two adaptive protocols. Finally, given the design of the study we cannot rule out a third possibility that the efficiency of a training protocol depends on the interaction of these two factors, initial amount of distortion and variability in distortion during training.

The current findings thus seem in line with the notion that initial exposure to "easy" items (not far removed from standard speech) facilitates learning. A previous study on auditory learning [22] and studies in the domain visual figure ground discrimination [51] and of sensory-motor adaptation (e.g., [52]) suggest that a gradual increase of the disparity between the new to-be-trained task conditions and the respective standard experience conditions allow for better adaptation to the "distortion" than a sudden introduction of distorted items. The findings that adaptive protocols are preferable to constant-difficult ones can be explained in terms of the Minimal Level Theory [45] or the Reverse Hierarchy Theory (RHT) of perceptual learning [53]According to the RHT, for example, speech perception relies on lexical (higher-level) and sensory (lower-level) representations [44]. Under adverse listening conditions, speech perception cannot rely on readily accessible lexical representations since these do not match the distorted acoustic input. Therefore, detailed sensory representations of the acoustic properties of the input that are not readily available need to be utilized, and this requires learning. By this account, during the first trials of adaptive training, as well as in training in the constant low-compression condition, the difference between the acoustic input and lexical representation is relatively small and this helps the learning system to gradually adapt to the increasing level of distortion in subsequent trials, and select the best representations of the distorted input that will subsequently consolidate and become a basis for deciphering increasingly distorted stimuli [45,51].

Although starting training with easy items may contribute to the perceptual learning of speech, our findings suggest that it does not guarantee better generalization. In the current study, the generalization of learning to new tokens was stronger in the adaptive training protocols than in the constant training procedures. The results of the new-token condition suggest that the knowledge obtained with training on the constant protocols is more dependent on the items to which participants were exposed to during training (i.e. improvements were more item or token specific). Indeed, in the adaptive procedures listeners encountered more speech-rate variability then in either of the constant procedures. Other studies also suggest that variability or diversity are important for efficient generalization (e.g., [54,55]). For example, Japanese speakers who were trained to discriminate between the English /r/ and /l/ categories improved more robustly under high variability (multiple talkers) compared with low variability conditions (single talker) [54]. We did not find differences between the two adaptive protocols even though the incremental protocol provided both an extended initial experience with "easy" items and variable stimulus rates over the course of training.

The lack of a clearly advantageous training protocol in the current study may reflect the specific conditions of the current design rather than a general rule. Future studies should just investigate the contribution of other protocol-related variables to the perceptual learning of speech. A comparison between active and passive training protocols including the manipulation of feedback is of interest because previous research suggests that relatively passive training conditions (implicit training without feedback) can in some cases elicit greater learning gains compared with active training conditions (explicit training with feedback) [56].

The current study suggests that procedures that start with relatively easy training are beneficial for the perceptual learning of speech. It points to the robustness of the perceptual learning of speech since all training protocols elicited significant learning (compared with an untrained control group). Our results provide some support for a possible benefit for skill transfer afforded by *variable* training on speech decoding. For the decoding of compressed speech, the current results suggest, some of the insights from the notion of "errorless" training [33] may better apply than the notion that experience with difficult items is a necessary condition for robust learning [38]. Focused studies are needed to determine whether gradual training regimes can facilitate speech perception in populations with perceptual deficits such as the hearing impaired, or in second language learners.

## Supporting information

S1 File Results of the training pre/posttest phases of the different groups. (XLSX)
